# Nonaqueous Capillary Electrophoretic Separation of Analogs of (24*R*)-1,24-Dihydroxyvitamin D_3_ Derivative as Predicted by Quantum Chemical Calculations

**DOI:** 10.3390/molecules28135055

**Published:** 2023-06-28

**Authors:** Błażej Grodner, Teresa Żołek, Andrzej Kutner

**Affiliations:** 1 Department of Biochemistry and Pharmacogenomics, Faculty of Pharmacy, Medical University of Warsaw, 1 Banacha, 02-097 Warsaw, Poland; 2 Department of Organic and Physical Chemistry, Faculty of Pharmacy, Medical University of Warsaw, 1 Banacha, 02-097 Warsaw, Poland; teresa.zolek@wum.edu.pl; 3 Department of Drug Chemistry, Faculty of Pharmacy, Medical University of Warsaw, 1 Banacha, 02-097 Warsaw, Poland; andrzej.kutner@wum.edu.pl

**Keywords:** nonaqueous capillary electrophoresis, density functional theory, vitamin D_3_ derivatives of calcipotriol, PRI-2201, PRI-2203, PRI-2204, PRI-2205, quantitative determination, validated analytical method

## Abstract

Nonaqueous capillary electrophoretic (NACE) separation was obtained of analogs of (24*R*)-1,24-dihydroxyvitamin D_3_ derivative (calcipotriol) as predicted by quantum chemical calculations supported by the density functional theory (DFT). Among the key electronic properties investigated, absolute values of the dipole polarizability and energy gap between HOMO and LUMO molecular orbitals of the analog molecules differ significantly for particular analogs, and there is a direct relationship with their electrophoretic migration time. These differences and relationships suggest that the structurally related analogs should be separable in the electrostatic field. Indeed, the robust, sensitive, and rapid NACE method was first developed for the identification and determination of the anticancer analog of calcipotriol (coded PRI-2205) and its process-related impurities (coded PRI-2201, PRI-2203, and PRI-2204) in organic and aqueous biological solutions. The direct relation between the calculated electronic properties of the analogs and the experimental electrophoretic migration time could be a promising prospect for theoretically predicting the electrophoretic separations.

## 1. Introduction

**Figure 1:**
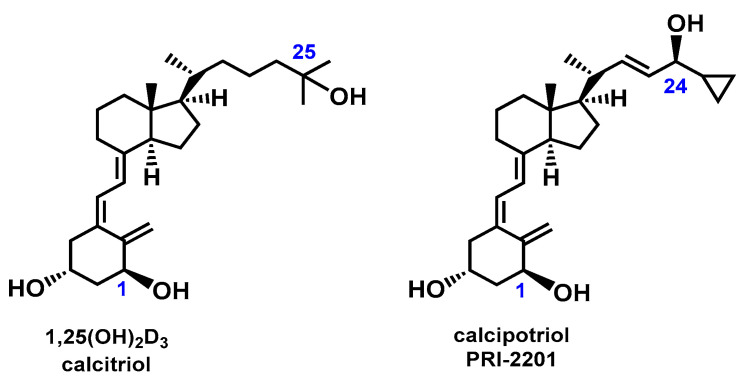
Structural formulas of 1,25-dihydroxyvitamin D_3_ [1,25(OH)_2_D_3_,calcitriol] and calcipotriol (PRI-2201).

Vitamin D_3_ is a fat-soluble vitamin that is synthesized in the skin from 5,7-dehydrocholesterol upon exposure to sunlight [1]. The biological effect of vitamin D_3_ is expressed through its interaction with the nuclear vitamin D receptor (VDR). The VDR is a tetrameric protein complex typical of all nuclear steroid receptors. Activation of the VDR leads to the activation of transcription factors [2,3,4,5,6]. Thus, the mechanism of interaction of the VDR—vitamin D_3_ complex with nuclear DNA is typical for steroid hormones [4,7]. The interaction of vitamin D_3_ with the membrane receptor in vitro was also reported. The final biological effect of vitamin D_3_ is caused by the transduction of the intracellular signal via second messengers [5,8,9]. The major biological function of vitamin D_3_ is to maintain normal blood levels of calcium and phosphorus. This is why vitamin D_3_ is necessary for mineral homeostasis and the proper formation of bone [10]. Vitamin D_3_ is reported to provide protection from and decrease an individual’s risk of developing osteoporosis, hypertension, cancer, and several autoimmune diseases. This is related to the stimulation of the body to produce antibodies and increase the strength of the immune system [11]. Vitamin D_3_ activates the production of anti-inflammatory factors, such as interleukin-4, and inhibits the formation of proinflammatory factors, such as interleukin 6, tumor necrosis factor, and macrophage colony-stimulating factor [12,13,14,15]. The neuroprotective role of vitamin D_3_ was also reported, regarding the reduction of the Ca^2+^ level in the brain [16,17,18,19,20,21] and the inhibition of brain γ-glutamyl transpeptidase [12,16]. The anticancer property of vitamin D has been studied in a wide variety of commonly occurring cancers (both in vitro and in vivo) of which colorectal, breast, and prostate cancers have been most responsive. Epidemiological studies provide strong evidence that the active form of vitamin D_3_, 1α,25-dihydroxyvitamin D_3_ [1,25(OH)_2_D_3_, calcitriol] (Figure 1), reduces the incidence of common human cancers, including carcinomas of the breast, prostate, and colon [22,23,24,25].

**Figure 2:**
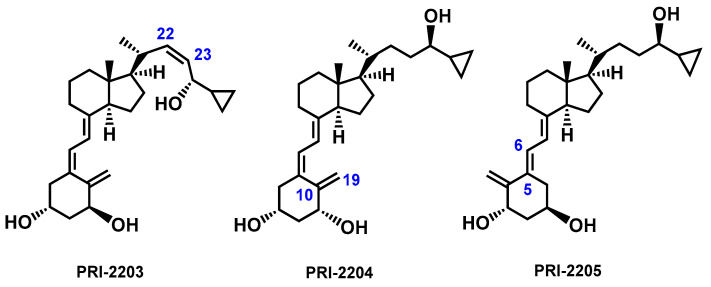
Structural formulas of analogs of calcipotriol, PRI-2203, PRI-2204, and PRI-2205.

The above information clearly shows that vitamin D_3_ plays a very important role in our lives, and therefore, research related to the function of vitamin D_3_ still arouses great interest. There are also high expectations associated with vitamin D_3_ analogs due to their potential therapeutic effect [26]. Analogs of calcipotriol, PRI-2201 (Figure 1) and PRI-2205 (Figure 2) showed antiproliferative activity in vitro against human cancer cell lines [27].

The analogs were more active than calcitriol and calcipotriol in terms of mouse Lewis lung cancer inhibition. Very advantageously, PRI-2205 revealed no undesired calcemic activity that limits the clinical use of the parent calcitriol. Although PRI-2205 potentiated the antiproliferative effect of *cis*-platin and doxorubicin against leukemia HL-60 cells and that of tamoxifen against MCF-7 breast cancer cells at higher doses than the reference calcitriol, it significantly increased VDR expression [28]. Additionally, PRI-2205 significantly enhanced the antitumor activity of 5-FU depending on the treatment regimen, decreased tumor growth and metastasis, and prolonged the survival time of research animals, compared to 5-FU alone [29]. The analogs PRI-2203 and PRI-2204 are representative process-related or degradation impurities of PRI-2205 [28].

To assess pharmacokinetic, pharmacological, and bioavailability parameters, there is a need to develop a method to confirm the identity and homogeneity of a given analog as well as one for their qualitative and quantitative determination, especially in biological material. Of great interest is a straightforward method for the separation of an analog from its diastereomers and geometric isomers as potential process-related impurities or degradation products. HPLC has mainly been reported [30,31,32,33,34] for the determination of vitamin D_3_ and metabolites in biological matrices and food products. However, none of these methods were used for synthetic analogs of calcitriol. Previously, we achieved the separation of vitamin D diastereomers by chiral stationary phase HPLC [35,36]. However, due to the sensitivity of chiral HPLC columns, these separations were limited to a few solvent systems and are not applicable to a broad range of synthetic vitamin D analogs.

At present, the effectiveness of separation is often based on experiments using capillary electrophoresis (CE). CE is a family of related techniques that employ narrow-bore capillaries to perform high efficiency separations of both large and small molecules. These separations are facilitated by the use of high voltages, which may generate electroosmotic and electrophoretic flow of buffer solutions and ionic species, respectively, within the capillaries. Under the influence of an electric field, the ionic species in a sample that is introduced as a zone into an electrolyte at one end of a capillary will be separated into discrete bands when they migrate to the other end of the capillary at different electrophoretic velocities. Capillary electrophoresis comprises a family of techniques that sometimes have extremely different operative and separative characteristics. Among these techniques, the following should be distinguished: capillary zone electrophoresis (CZE), isoelectric focusing, capillary gel electrophoresis, isotachophoresis, and micellar electrokinetic capillary chromatography. Nonaqueous capillary electrophoresis (NACE) is a variant of CZE that uses organic solvents, which are the main components of solutions in which the separation of compounds that are sparingly soluble or insoluble in polar solvents is carried out. The main benefit of using organic solvents instead of water in CE is the improvement of analyte solubility, especially when UV detectors are used. In many cases, the use of organic solvents leads to significantly shorter analysis times and a higher separation efficiency [37]. To the best of our knowledge, the NACE method has not been used to study vitamin D analogs so far.

However, experimental methods are labor-intensive and expensive. For this reason, analog synthesis is being optimized using new methods, such as quantum mechanical calculations [38,39]. In recent years, quantum chemical (QC) methods have emerged as an efficient tool for the accurate prediction of stability and chemical reactivity of molecules [40,41,42]. Among the full methods used in QC, density functional theory (DFT) can mainly be mentioned. Due to its theoretical nature, DFT is considered a “green technique” for optimizing chemical structures. Various QC parameters, such as the dipole moment (μ), dipole polarizability (α), energy difference, highest occupied molecular orbital (E_HOMO_), and lowest occupied molecular orbital (E_LUMO_), etc., are used to determine the molecular properties of newly designed compounds. The global molecular properties also justify the reactivity and stability of synthetic calcitriol analogs. The most stable analog structure has the largest HOMO-LUMO energy gap. Therefore, an electron system with a larger HOMO-LUMO energy gap is less reactive than one with a smaller gap. Moreover, according to the principle of minimum polarizability of an electric dipole, the natural direction of the evolution of a given structure is the state of minimum polarizability [43].

In this paper, we present a combination of computational and experimental techniques to investigate the possible relationship between the molecular structures and electronic properties of a series of structurally related vitamin D_3_ analogs and their electrophoretic separation. Here, we report the method for their simultaneous determination using nonaqueous capillary electrophoresis (NACE) and quantum chemical parameters calculated by the DFT method.

## 2. Results and Discussion

### 2.1. Electronic Properties of Vitamin D_3_ Analogs

To investigate the effect of the molecular structure on the mobility of analogs PRI-2201, PRI-2203, PRI-2204, and PRI-2205 in the electrostatic field, full-structure optimization was first performed for each molecule. All categories of the electronic properties of the analogs, including the dipole moment, dipole polarizability, the energies of the HOMO and LUMO molecular orbitals, and the molecular electrostatic potential (MEP) were calculated (Table 1, Figure 4 and Figure 5, and Tables S1 and S2 in the Supplementary Materials).

**Figure 3:**
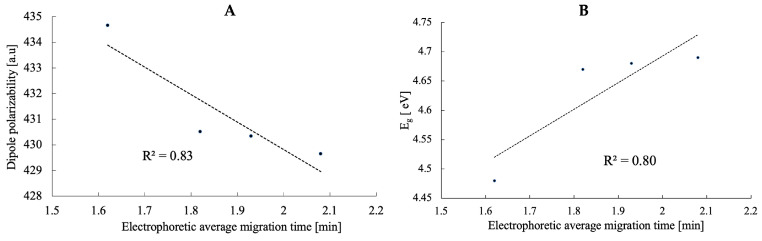
The linear correlation between electrophoretic average migration time *vs.* dipole polarizability (**A**) and HOMO-LUMO energy gap (**B**) of analogs of calcipotriol.

The molecular dipole moment is a basic property of a molecule that is used to investigate intermolecular interactions. An increase in the dipole moment raises the polar nature of a bond in a molecule. The DFT-calculated data indicate that the dipole moment of four vitamin D_3_ analogs was in the order PRI-2204 < PRI-2203 < PRI-2205 < PRI-2201. The highest dipole moments of PRI-2201 and PRI-2205 signified their strong intermolecular interactions. The dipole moment for PRI-2201 (μ = 4.23 Debye), larger than that of PRI-2205 (μ = 3.49 Debye), may be due to the presence of a strong electron donor–acceptor configuration in the molecule. As we noted earlier, dipole polarizability is a quantity of particular interest for structural chemistry. The molecular polarizability of a molecule characterizes the capability of its electronic system to be distorted by the external field, and it plays an important role in modeling many molecular properties and biological activities [44]. This parameter relates to how the susceptibility of system electrons can be influenced by the approaching charge. This is affected by several factors, such as the compound complexity, the size of the molecular systems, and the number of free electrons. Herein, the order of mean polarizability PRI-2205 > PRI-2204 > PRI-2203 > PRI-2201 suggests that the molecule PRI-2205 (α_total_ = 434.66 a.u.) is the most polarizable; regardless, their differences are not very high. In addition, the theoretically determined dipole polarizability values were well correlated with the experimentally determined average electrophoretic migration times for a small set of data with four molecules (R^2^ = 0.83, Figure 3A), only. The correlation coefficient was high enough, proving the model’s predictive ability. Thus, the model is suitable for predicting the order of migration of vitamin D_3_ analogs in NACE.

Another key electronic property investigated was the study of the HOMO and LUMO distribution in the molecules to assess their electron delocalization and chemical reactivity. The energy gap (E_g_ = E_HOMO_-E_LUMO_) explains the charge transfer interaction occurring within the molecules. HOMO represents the ability to donate an electron, and LUMO means the ability to obtain an electron. The E_g_ reveals the chemical reactivity of molecules and proves the occurrence of charge transfer within molecules. The frontier orbital gap helps to characterize the molecules’ chemical reactivity and kinetic stability. A molecule with a small frontier orbital gap is more polarizable. It is generally associated with a high chemical reactivity and low kinetic stability and is also termed a soft molecule [45]. The calculated HOMO and LUMO gaps, the E_g_ values, are in the narrow range of −4.48 to −4.69 eV (Table 1). The PRI-2205 molecule has the lowest E_g_ (−4.48 eV), suggesting it has the highest polarizability. The E_g_ values increase from −4.67 eV for PRI-2204 through to−4.68 eV for PRI-2203 and−4.69 eV for PRI-2201. Similarly, the electrophoretic mobility changes from 1.82 min for PRI-2204 to 1.93 min for PRI-2203 and 2.08 min for PRI-2201. The correlation between the E_g_ value and the experimental electrophoretic average migration time (R^2^ = 0.80, Figure 3B)confirms that structural differences affect the migration of analytes.

**Figure 4:**
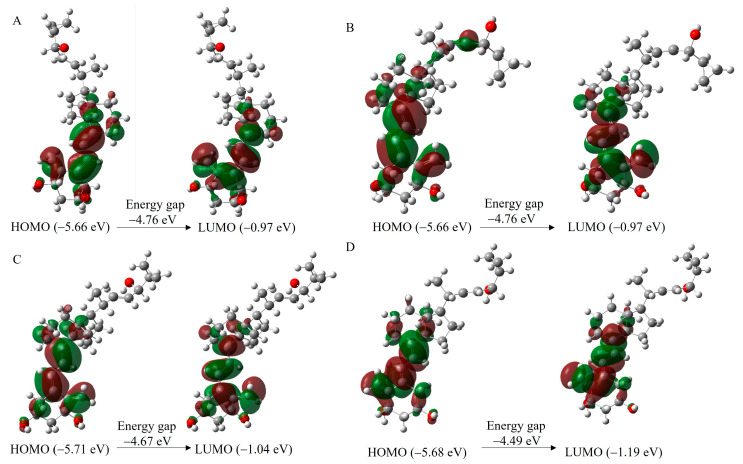
HOMO-LUMO diagram of PRI-2201 (**A**), PRI-2203 (**B**), PRI-2204 (**C**), and PRI-2205 (**D**). The positive (red) and negative (green) phase distributions represent the molecular orbital wave functions. HOMO (electron donor regions) determines the ionization potential. LUMO (electron acceptor regions) determines the electron affinity.

For all investigated molecules, the isosurfaces of molecular orbitals and electron density differences were delineated (Figure 4).The charge distribution in the HOMOs is localized on the A-ring hydroxyls, 19-methylene at C-10, at the C-5 position in the vitamin D triene system, and on the side chain for PRI-2203, whereas the distribution of LUMOs occurs in 19-methylene at C-10 and in the triene system at C-5.

**Figure 5:**
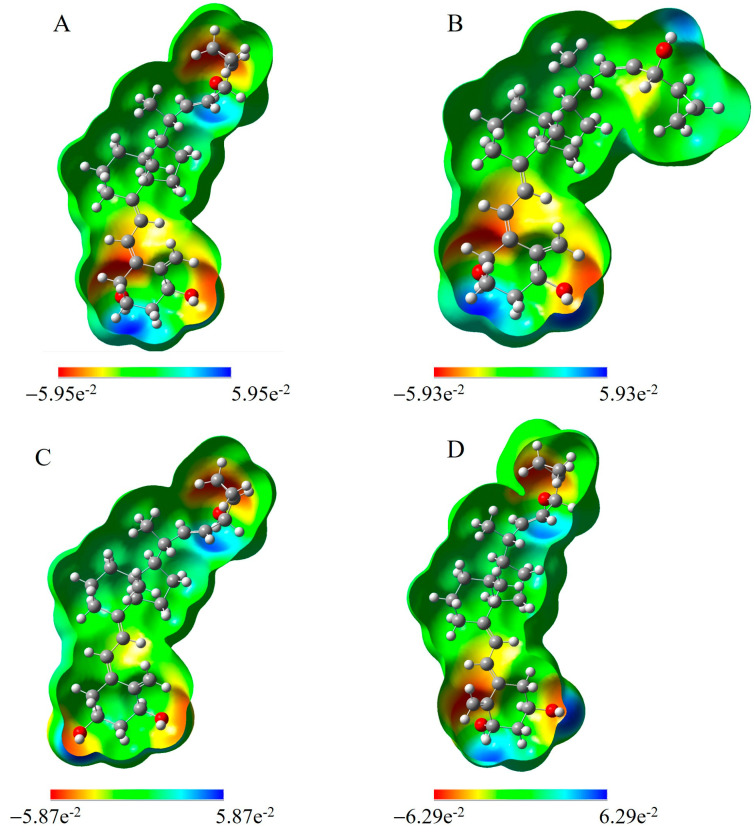
Molecular electrostatic potential (MEP) maps of PRI-2201 (**A**), PRI-2203 (**B**), PRI-2204 (**C**), and PRI-2205 (**D**). The MEP represents electron-rich (red) and electron-poor (blue) regions.

Moreover, for all analogs, we calculated molecular electrostatic potential (MEP) maps(Figure 5).The MEP is an important descriptor to validate the reactivity related to the molecular system. It is also an important tool to envisage how molecules with different geometries can interact, and how the charge can be transferred. The three-dimensional MEP surface gives an indication of the position, shape, and size of the positive, negative, and neutral electrostatic potentials. The maximum positive region is the preferred site for the nucleophilic attack, which is designated by blue color. Likewise, a maximum negative region is favored for an electrophilic attack, represented by a red surface. The proton-rich region exhibits positive potential, and the repulsion energy between the protons is marked as the green region. For all analogs, high electrostatic potential regions are present in the vicinity of the hydroxyl’s oxygen. A low electropositive potential is mainly found on the hydrogen atoms of hydroxyls present in the A-ring. Further, the variance in the MEP surface shows an increase in positive potential due to the proton environment in the molecule.

### 2.2. Development of Capillary Electrophoresis

The NACE method was developed for the determination of analogs of the derivative of (24*R*)-1,24-dihydroxyvitamin D_3_. The type and composition of the separation electrolyte (background electrolyte—BGE) were studied in different concentrations of sodium acetate and methanol and with different acetonitrile ratios in the separation solution for the quantification of the analogs PRI-2205,PRI-2204, PRI-2203, and PRI-2201.

The effect of the sodium acetate concentration was studied in the range of 50 to 200 mM for the BGE containing methanol only. When the concentration of sodium acetate was 50 mM, the peak shapes were inadequate, and the resolution for all investigated compounds was not obtained (Figure S1A included in the Supplementary Materials). Therefore, by varying the concentration, the best resolution was achieved. The resolution of the investigated compounds increased with an increasing concentration of sodium acetate in BGE. A clear but only partial separation of PRI-2205, PRI-2204, PRI-2203, and PRI-2201 was obtained at a sodium acetate concentration of 100 mM (Figure S1B included in the Supplementary Materials). Determination of the analogs was carried out further by increasing the concentration of sodium acetate. Increasing the sodium acetate concentration to 200 mM negatively affected the resolving power of BGE (Figure S1C included in the Supplementary Materials). Finally, the best primary resolution was obtained with a sodium acetate concentration of 100 mM (Figure S1B included in the Supplementary Materials).

Then, in order to increase the resolution, the quantitative composition of the methanol:acetonitrile mixture in the BGE was modified. The initial separation of the PRI-2205, PRI-2204, PRI-2203, and PRI-2201 analogs was carried out in plain methanol and at a sodium acetate concentration of 100 mM (Figure S2A included in the Supplementary Materials). Improvement of the resolution was obtained after increasing the content of acetonitrile to the BGE. The use of a mixture of methanol:acetonitrile at a ratio of 90:10 significantly improved the resolution (Figure S2B included in the Supplementary Materials). However, the best resolution was obtained using a mixture of methanol:acetonitrile at a ratio of 80:20 and a sodium acetate concentration of 100 mM (Figure S2C included in the Supplementary Materials).

Various analytical conditions were also examined. The voltage, analysis temperature, capillary length, and detection wavelength were optimized in the ranges of 5–20 kV, 20–30 °C, 30–80 cm (total length), and 200–300 nm, respectively. The greatest influences on the detection, separation, and analysis time of PRI-2205, PRI-2204, PRI-2203 and PRI-2201 came from the wavelength, current voltage, and capillary length. The best detection was obtained at a wavelength of 273 nm, providing maximal absorption of 5,6-*trans* analogs of vitamin D, such as PRI-2205. The best resolution and the shortest analysis time were obtained at a voltage of 10 kV. Further increasing the voltage value accelerated the analysis time but decreased the resolution and stability of the current intensity. The length of the capillary also had significant effects on the resolution and analysis time. The use of the longest capillary resulted in very good separation, but the analysis time was very long. With the use of shorter capillaries, the migration time between individual analogs was shortened, together with the analysis time. However, the use of the shortest capillary maintained appropriate resolutions for all four vitamin D_3_ analogs, and the separation was obtained in the shortest analysis time. Ultimately, the best separation parameters were obtained using a separation solution of 80:20 (methanol: acetonitrile) containing sodium acetate at a concentration of 100 mM at 273 nm, 20 °C, and 10 kV with a capillary length of 30 cm (Figure S1 is included in the Supplementary Materials).

**Figure 6:**
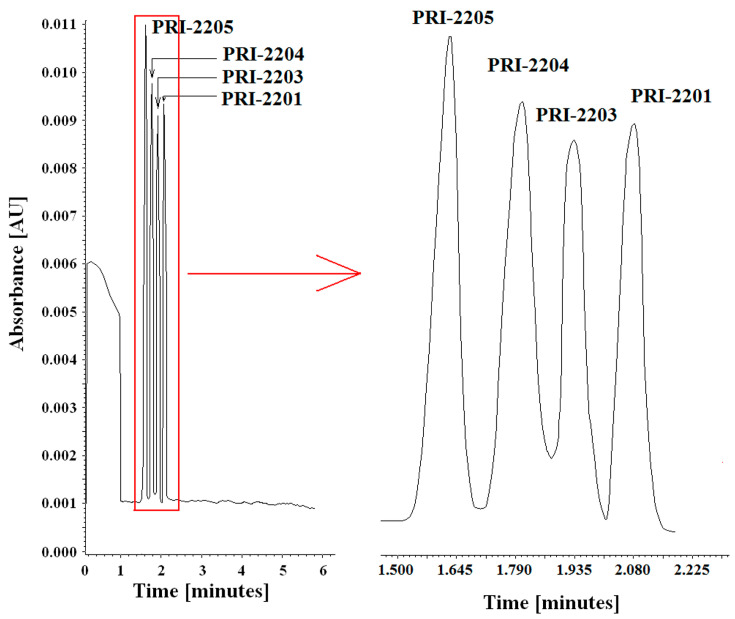
Electropherograms of the mixture of analogs, PRI-2205, PRI-2204, PRI-2203, and PRI-2201, in 50 mM of sodium acetate and various ratios of methanol:acetonitrile mixture: 50 mM sodium acetate in 80:20 methanol:acetonitrile mixture.

To determine the selectivity of the method, electropherograms of extracts of blank serum samples and serum samples containing PRI-2205, PRI-2204, PRI-2203, and PRI-2201 were analyzed. In the absence of interference caused by the presence of endogenous compounds, the method was defined as selective. The specificity of the method was determined by comparing electropherograms of the blank solution, solutions containing PRI-2205, PRI-2204, PRI-2203, and PRI-2201, blank serum samples, and serum samples containing analogs. In all cases, no interference was observed between the peaks from endogenous substances and the migration times of the tested analogs (Figure 6 and Figure S2 is included in the Supplementary Materials).

## 3. Materials and Methods

### 3.1. Quantum Chemical Calculations

Theoretical calculations were performed using the Gaussian 16W suite of software [46]. The starting conformations of PRI-2205, PRI-2204, PRI-2203, and PRI-2201 were constructed based on the solid-state diffraction data of structurally related compounds to eliminate any subjectivity in generating the three-dimensional structure [26]. The molecular structures of the analogs were optimized using density functional theory (DFT) with the Becke-3-parameter–Lee–Yang–Parr (B3LYP) hybrid functional [47] and the 6-311G(d,p) basic set [48]. The solvent effect was considered using the Polarizable Continuum Model (PCM) implemented in the Gaussian 16W. Methanol was used in all calculations considering the experimental conditions. Vibrational frequency calculations ensured that the obtained structures represented the global minima. All calculations were performed using standard gradient techniques and default convergence criteria. The quantum chemical parameters, such as the E_HOMO_ (energy of the highest occupied molecular orbital), E_LUMO_ (energy of the lowest unoccupied molecular orbital), E_g_ (energy gap), dipole moment (μ), and dipole polarizability (α) were calculated to reveal the compounds’ stability and chemical reactivity. The electrostatic potential (ESP) of the atomic partial charges on the atoms was computed using the Breneman model [49], which reproduces the molecular electrostatic potential.

### 3.2. Chemicals and Reagents

The synthesis, spectroscopic analysis (NMR and MS), and biological activity of PRI-2205, PRI-2204, PRI-2203, and PRI-2201 were described previously [27,28,29]. The purities of the reference compounds were found by HPLC to be greater than 98%. All other analytes were of a purity of 99%. Acetonitrile, methanol, sodium acetate, and human serum standard were obtained from Sigma-Aldrich (Poznań, Poland).

### 3.3. Instrumentation

A Capillary Electrophoresis Beckman Coulter P/ACE MDQ system, equipped with an autosampler and a UV/Visible detector, was used. All the parameters of the CE were controlled by Karat software version 32.

### 3.4. CE Conditions

The measurements were performed using a Beckman capillary electrophoresis system with a UV array detector. An amine permanently coated fused-silica eCAP capillary with an inner diameter of 75 μm, total length of 40 cm, and efficient length of 30 cm was used. An eCAP fused-silica capillary (30 cm total length, 20 cm effective length, 50 μm id, 375 μm od) was also used. The capillaries were kept at 20 °C. Detection was performed at 273 nm. Hydrodynamic sample injection was performed under a pressure of 3 psi in 5 s. Prior to use, the capillary was washed with a 0.1 M NaOH solution for 5 min, then with water for 5 min, and with a background electrolyte solution for 10 min. Between the analyses, it was washed with a background electrolyte solution (100 mM sodium acetate in methanol:acetonitrile 80:20) for 3 min. The separation was carried out at 10 kV.

### 3.5. Preparation of Stock and Working Standard Solutions

Primary standard stock solutions for the analogs PRI-2201, PRI-2203, PRI-2204, and PRI-2205 were prepared separately by dissolving 100 μg of each analog in 1 mL of methanol. These solutions were further diluted with methanol to obtain working standard solutions of 10.00 μg/mL, 20.00 μg/mL, 40.00 μg/mL, 80.00 μg/mL, 160.00 μg/mL, and 320.00 μg/m.

To prepare the calibration curve, 25 µL of each of the working solutions was taken and supplemented with methanol to a volume of 1 mL to obtain mixtures of all analogs with final concentrations of 0.25, 0.50, 1.00, 2.00, 4.00, and 8.00 μg/mL. The serum solutions were obtained similarily. For this purpose, 25 mL of each of the analog working solutions was added to six test tubes containing 0.975 mL of serum, thus obtaining final concentrations of 0.25, 0.50, 1.00, 2.00, 4.00, and 8.00 μg/mL for each analog. All samples were thoroughly vortexed for 5 min. Then, 3 mL of 80:20 hexane:ethyl acetate was added to each sample and thoroughly vortexed again for 5 min. The mixture was centrifuged (5 min, 1000 g), and the organic layer was transferred to a separate tube. This operation was repeated. Then, the test tubes with the extracted analogs and the hexane:ethyl acetate mixture were evaporated in a stream of nitrogen at 37 °C. The residues were dissolved in 1 mL of methanol and injected into the capillary.

### 3.6. Method Validation

A description and the validation conditions of the method are included in the “Supplementary Materials” section.

## 4. Conclusions

Electrophoretic separation of diastereomeric and isomeric vitamin D analogs was first predicted theoretically by calculating the electronic properties of the molecules and then obtained experimentally. The average electrophoretic migration time correlated best with the dipole polarizability. Rapid nonaqueous capillary electrophoresis (NACE) allowed for the identification and accurate, precise, and sensitive quantification of the structurally related analogs PRI-2205 and PRI-2204 and PRI-2203, and PRI-2201, which are associated with anticancer activity in organic solutions and serum. The extraction method used showed good linearity for all tested analogs (R^2^ > 0.9976). The method was suitable for determining analogs within an LOQ range of 287.1–310.1 ng/mL for the nominal analyte concentration. In addition, the RSD for intra- as well as interday precision was low and did not exceed 5% for all analogs. Further, to examine the scope and limitations of the predictive power of the calculated dipole polarizability for electrophoretic separation, closely related analogs of active forms of not only vitamin D_3_ but also D_2_ will be examined.

## Figures and Tables

**Table 1:** Calculated values for the dipole moment (μ) and dipole polarizability (α) and the average experimental electrophoretic migration times for PRI-2201, PRI-2203, PRI-2204, and PRI-2205.

Analogs	Hybrid Functional B3LYP 6-311 (d,p) in Methanol					Average Electrophoretic Migration Time ^a^ [min]
	μ_total_	α_total_	E_LUMO_	E_HOMO_	E_g_	
	[Debye]	[a.u.]	[eV]	[eV]	[eV]	
PRI-2201	4.23	429.65	−0.97	−5.66	−4.69	2.08 ± 0.07
PRI-2203	3.14	430.34	−0.97	−5.66	−4.68	1.93 ± 0.07
PRI-2204	3.09	430.51	−1.04	−5.71	−4.67	1.82 ± 0.05
PRI-2205	3.49	434.66	−1.19	−5.68	−4.48	1.62 ± 0.02

^a^ Average from *n* = 6 experiments.

## Data Availability

All relevant data are provided in the article.

## Supplementary Materials

The following supporting information can be downloaded at: https://www.mdpi.com/article/10.3390/molecules28135055/s1, Table S1.The DFT calculated total electric dipole moments, μ (Debye) and dipole moment components for PRI-2201, PRI-2203, PRI-2204, and PRI-2205.; Table S2. The DFT calculated total dipole polarizability and some selected components of the dipole polarizability for PRI-2201, PRI-2203, PRI-2204, and PRI-2205.; Table S3. Regression equation, limits of detection, and quantification for the PRI-2205, PRI-2204, PRI-2203, and PRI-2201 analogs (n = 6).; Table S4. Recovery data for the PRI-2205, PRI-2204, PRI-2203, and PRI-2201 vitamin D_3_ analogs from serum samples after the extraction procedure (n = 6). Table S5. Intraday and interday precision of the PRI-2205, PRI-2204, PRI-2203, and PRI-2201 analogs.; Figure S1. Electrophoregrams of the mixture of PRI-2205, PRI-2204, PRI-2203, and PRI-2201 analogs in different separation phase solutions: (A) 50 mM sodium acetate in methanol, (B) 100 mM sodium acetate in methanol, (C) 200 mM mM sodium acetate in methanol.; Figure S2. Electropherograms of the mixture of PRI-2205, PRI-2204, PRI-2203, and PRI-2201 analogs in 50 mM sodium acetate and different proportions of the methanol:acetonitrile mixture: (A) 50 mM sodium acetate in methanol, (B) 50 mM sodium acetate in 90:10 methanol:acetonitrile.
