# Supplementary material for: Nonaqueous Capillary Electrophoretic Separation of Analogs of (24R)-1,24-Dihydroxyvitamin D3 Derivative as Predicted by Quantum Chemical Calculations
Source: Molecules. 2023 Jun 28;28(13):5055. doi: 10.3390/molecules28135055 (PMC10343829; doi:10.3390/molecules28135055)
Supplement: Supplementary file 1 [file molecules-28-05055-s001.zip › molecules-2431563-supplementary.pdf]

Supplementary materials

Nonaqueous capillary electrophoretic separation of analogs of (24*R*)-1,24-dihydroxyvitamin D_3 derivative as predicted by quantum chemical calculations

Błażej Grodner [1,†,*], Teresa Żołek [2,†], Andrzej Kutner [3]

[1] Department of Biochemistry and Pharmacogenomics, Faculty of Pharmacy, Medical University of Warsaw, 1 Banacha, 02-097 Warsaw, Poland

[2] Department of Organic and Physical Chemistry, Faculty of Pharmacy, Medical University of Warsaw, 1 Banacha , 02-097 Warsaw, Poland; teresa.zolek@wum.edu.pl

[3] Department of Drug Chemistry, Faculty of Pharmacy, Medical University of Warsaw, 1 Banacha 0 2-097 Warsaw, Poland; andrzej.kutner@wum.edu.pl

* Correspondence: blazej.grodner@wum.edu.pl

† Both authors contributed equally to this work.

Table S1. The DFT calculated total electric dipole moments, μ (Debye), and dipole moment components for PRI-2201, PRI-2203, PRI-2204, and PRI-2205.

Analogs	Hybrid functional B3LYP 6-311 (d,p) in methanol			
	μ_x	μ_y	μ_z	μ_{total}
PRI-2201	-3.5997	0.3283	2.1765	4.2194
PRI-2203	-3.0681	-0.2957	-0.5817	3.1367
PRI-2204	-0.7882	0.6409	2.9258	3.0971
PRI-2205	-2.1445	-0.0851	2.7613	3.4973

Table S2. The DFT calculated total dipole polarizability and some selected components of the dipole polarizability for PRI-2201, PRI-2203, PRI-2204, and PRI-2205.

Analogs	Hybrid functional B3LYP 6-311 (d,p) in methanol			
	α_{xx}	α_{yy}	α_{zz}	α_{total}
PRI-2201	389.626	427.316	472.009	429.650
PRI-2203	337.044	454.564	499.415	430.341
PRI-2204	509.650	430.323	351.564	430.513
PRI-2205	439.551	417.346	447.081	434.659

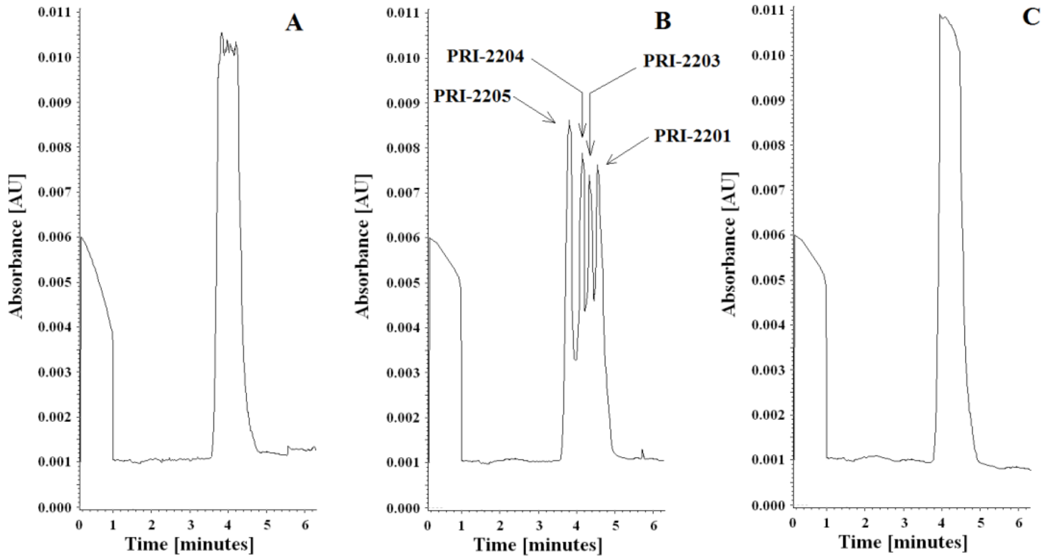

Figure S1. Electrophoregrams of the mixture of PRI-2205, PRI-2204, PRI-2203 and PRI-2201 analogs in different solutions of separation phase: (A) 50 mM sodium acetate in methanol, (B) 100 mM sodium acetate in methanol, (C) 200 mM mM sodium acetate in methanol.

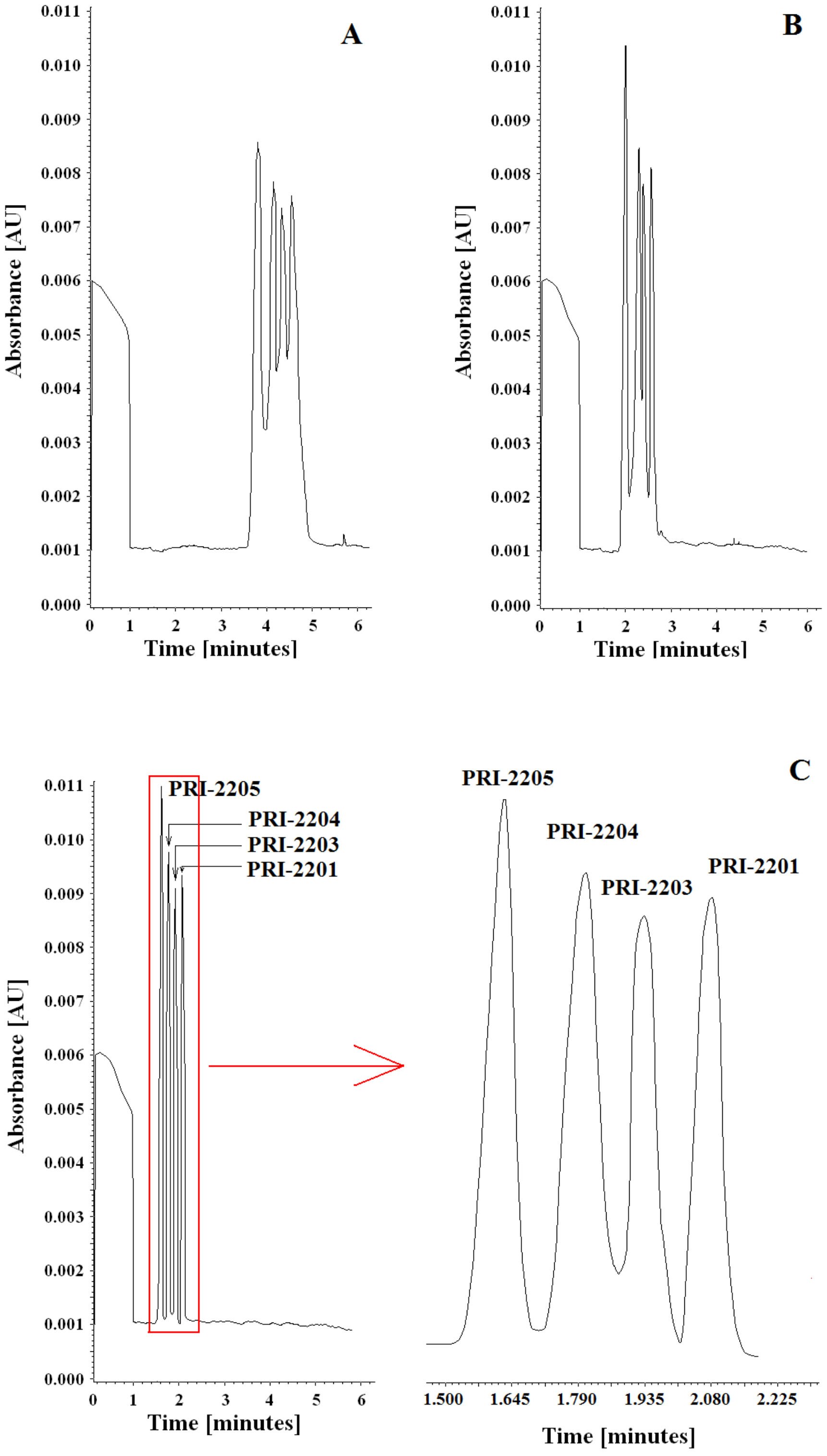

Figure S2. Electropherograms of the mixture of PRI-2205, PRI-2204, PRI-2203 and PRI-2201 analogs in 50 mM sodium acetate and different proportions of methanol : acetonitrile mixture: (A) 50 mM sodium acetate in methanol, (B) 50 mM sodium acetate in 90:10 methanol : acetonitrile, (C) 50 mM sodium acetate in 80:20 methanol : acetonitrile mixture.

S1.1. Method validation

The methodology for analysis of PRI-2205, PRI-2204, PRI-2203 and PRI-2201 analogs using NACE was validated in terms of selectivity, linearity, sensitivity, precision, and accuracy according to the ICH guidelines Q10 [43]. The linearity of the method was tested in the concentration range of 0.25-8.00 µg/mL by preparing six calibration curves on six consecutive days. As described earlier (in chapter 2.6) 25 ml of each of the standard solutions of I, II, III and IV analogs were added to six test tubes containing 0.975 ml of serum, thus obtaining final concentrations of 0.25, 0.50, 1.00, 2.00, 4.00 and 8.00 µg/mL for each analog, respectively. The correlation coefficients of calibration curves were in the range of 0.9976 – 0.9998 for all four analogs (Table S3). LOD and LOQ were determined at S/N (of signal-to-noise ratio) of 3 and 10, respectively.

Table S3. Regression equation, limits of detection, and quantification for PRI 2205, PRI 2204, PRI 2203, and PRI 2201 analogs (n=6).

Compounds	Linearity range µg/mL	R^2	RSD (%)	LOD µg/mL	LOQ µg/mL	Regression equation	Standard deviation Slope	Intercept
PRI-2205	0.25 – 8.0	0.9998	3.26	0.085	0.25	y=39.984x-0.203	±2.437	±0.062
PRI-2204	0.25 – 8.0	0.9979	4.13	0.092	0.25	y=39.688x+0.018	±2.648	±0.005
PRI-2203	0.25 – 8.0	0.9976	4.45	0.089	0.25	y=39.679x-0.170	±3.014	±0.047
PRI-2201	0.25 – 8.0	0.9998	3.24	0.101	0.25	y=40.061x-0.040	±2.029	±0.006

S1.2. Limit of detection and quantification

The limits of detection (LOD) and limits of quantification (LOQ) were determined considering the corresponding concentration to produce a signal 3 and 10 times, respectively, the baseline noise in a close region to the migration time of each compound. The LOD was determined by analyzing loaded serum samples with increasing analyte content. Each concentration was analyzed six times. The proposed method allows the PRI-2205, PRI-2204, PRI-2203, and PRI-2201 analogs to be determined with LODs between 0.085–0.101 µg/mL and LOQs between 0.277–0.329 µg/mL (Table S3).

S1.3. Absolute recovery

Analytical recoveries were performed at three different concentrations (0.25, 1.000, and 4.000 µg mL, for all four analogs). The recovery of the extraction procedure was determined by comparing the values of peak areas obtained with those of standard

solutions of equivalent concentrations of five of the analytes. The recovery of concerned analytes was found in a satisfactory range of 94.7–99.0% (Table S4).

Table S4. Recovery data of PRI-2205, PRI-2204, PRI-2203, and PRI-2201 vitamin D_3 analogs from serum samples after extraction procedure (n = 6).

Vitamin D_3 analog	Added amount µg/mL	Observed amount µg/mL	%Recovery	% RSD
PRI-2205	0.25	0.24±0.008	95.6	3.33
	1.0	0.98±0.035	98.0	3.57
	8.0	7.96±0.050	99.5	1.26
PRI-2204	0.25	0.23±0.009	94.1	3.83
	1.0	0.96±0.037	96.5	3.85
	8.0	7.86±0.071	98.3	1.81
PRI-2203	0.25	0.23±0.009	92.4	3.90
	1.0	0.93±0.038	93.4	4.09
	8.0	7.78±0.082	97.3	2.11
PRI-2201	0.25	0.24±0.008	95.2	3.35
	1.0	0.97±0.035	97.0	3.61
	8.0	7.94±0.056	99.3	1.41

S1.4. Intra and inter-day precision

The precision and accuracy of validation data are summarized in Table S3. The intra-day precision was within limits of 2.3 % - 3.6 % RSD for the retention time and 2.7 % - 4.3 % RSD for the peak area. The inter-day precision was within limits of 2.0 % - 3.3 % RSD for the retention time and 2.8 % - 4.2 % RSD for the peak area for all five investigated compounds. The precision results (Table S5) showed the low values of intra- and interday % RSD of retention times and peak areas (<4.3 %).

Table S5. Intra-day and inter-day precision of compounds PRI-2205, PRI-2204, PRI-2203, and PRI-2201 analogs.

Vitamin D_3 analog	Concentration [μg/mL]	Intra-day precision (n=6, mean) %RSD						Inter-day precision (n=18, mean) %RSD	
		Day-1		Day-2		Day-3			
		t_R[a]	p_A[b]	t_R[a]	p_A[b]	t_R[a]	p_A[b]	t_R[a]	p_A[b]
PRI-2205	0.25	2.5	4.7	2.4	4.8	2.4	4.7	2.6	4.8
	1.00	1.9	3.8	1.8	3.8	1.8	3.6	2.0	3.7
	4.00	1.3	2.4	1.3	2.6	1.4	2.5	1.4	2.7
PRI-2204	0.25	2.7	4.9	2.8	4.8	2.9	4.8	3.0	4.9
	1.00	2.1	3.9	2.0	3.9	2.0	3.8	2.2	3.8
	4.00	1.4	2.8	1.5	2.9	1.5	2.9	2.0	3.0
PRI-2203	0.25	2.8	5.1	2.9	4.9	2.8	5.1	2.9	5.1
	1.00	2.3	4.1	2.2	4.0	2.1	3.9	2.4	4.0
	4.00	1.6	3.2	1.6	3.1	1.8	3.2	1.8	3.3
PRI-2201	0.25	2.6	4.8	2.5	4.8	2.6	4.9	2.7	4.9
	1.00	1.9	3.9	1.9	3.8	2.0	3.9	2.1	3.9
	4.00	1.4	2.8	1.3	2.7	1.4	2.9	1.5	2.9

[a]% RSD of retention time; [b]% RSD of peak area
